# Involvement of CREB-regulated transcription coactivators (CRTC) in transcriptional activation of steroidogenic acute regulatory protein (*Star*) by ACTH

**DOI:** 10.1016/j.mce.2019.110612

**Published:** 2020-01-01

**Authors:** Lorna I.F. Smith, Victoria Huang, Mark Olah, Loc Trinh, Ying Liu, Georgina Hazell, Becky Conway-Campbell, Zidong Zhao, Antoine Martinez, Anne-Marie Lefrançois-Martinez, Stafford Lightman, Francesca Spiga, Greti Aguilera

**Affiliations:** a Section on Endocrine Physiology, Program on Developmental Endocrinology and Genetics, Eunice Kennedy Shriver National Institute of Child Health and Human Development, NIH, Bethesda, MD, USA; b Henry Wellcome Laboratories for Integrative Neuroscience and Endocrinology, University of Bristol, Bristol, UK; c Génétique Reproduction & Développement, CNRS UMR 6293, Inserm U1103, Université Clermont Auvergne, 63001, Clermont-Ferrand, France

**Keywords:** CRTC, StAR, CREB, Steroidogenesis, Adrenal cortex, Transcription

## Abstract

Studies *in vivo* have suggested the involvement of CREB-regulated transcription coactivator (CRTC)2 on ACTH-induced transcription of the key steroidogenic protein, Steroidogenic Acute Regulatory (StAR). The present study uses two ACTH-responsive adrenocortical cell lines, to examine the role of CRTC on *Star* transcription. Here we show that ACTH-induced *Star* primary transcript, or heteronuclear RNA (hnRNA), parallels rapid increases in nuclear levels of the 3 isoforms of CRTC; CRTC1, CRTC2 and CRTC3. Furthermore, ACTH promotes recruitment of CRTC2 and CRTC3 by the *Star* promoter and siRNA knockdown of either CRTC3 or CRTC2 attenuates the increases in ACTH-induced *Star* hnRNA. Using pharmacological inhibitors of PKA, MAP kinase and calcineurin, we show that the effects of ACTH on *Star* transcription and CRTC nuclear translocation depend predominantly on the PKA pathway. The data provides evidence that CRTC2 and CRTC3, contribute to activation of *Star* transcription by ACTH, and that PKA/CRTC-dependent pathways are part of the multifactorial mechanisms regulating *Star* transcription.

## Highlights

• ACTH induces CRTC1-3 translocation to the nucleus in Y1-BS1 and ATC7-L cells.
• This parallels increases in pCREB and precedes initiation of *Star* transcription.
• CRTC2 and CRTC3 bind the *Star* promoter during transcriptional activation.
• Inhibition or knockdown of CRTC1-3 attenuates ACTH-induced *Star* transcription.
• Implicates role for CRTC 2 and 3 in multifactorial regulation of *Star* transcription.

## Abbreviations

CBD: CREB binding domain
CBP: CREB binding protein
ChIP: chromatin immunoprecipitation
CRE: cyclic AMP response element
CREB: cyclic AMP response element binding protein
CRTC: CREB-regulated transcription coactivator
CsA: cyclosporine A
EGF: epidermal growth factor
hnRNA: heteronuclear RNA
MAPK: mitogen-activated protein kinase
MC2R: melanocortin 2 Receptor
MRAP: MC2R accessory protein
mRNA: messenger RNA
pCREB: phosphorylated cyclic AMP response element binding protein
pen/strep: penicillin and streptomycin
PKA: protein kinase A
PMA: phorbol 12-myristate 13-acetate
siRNA: silencing RNA
StAR: steroidogenic acute regulatory protein
SF-1: steroidogenic factor 1

## 1 Introduction

Glucocorticoid hormone release from the adrenal gland is essential for maintaining normal metabolic function and survival during severe stress (Cole et al., 1995; Whitehead et al., 2013). In basal (unstressed) conditions, glucocorticoids are secreted rhythmically, with both circadian (daily) and ultradian (pulsatile) variations (Park et al., 2013; Weitzman et al., 1971; Tapp et al., 1984). The episodic nature of glucocorticoid secretion is critical for homeostasis, and its alteration can result in changes in tissue steroid responsiveness, resulting in neuroendocrine, behavioural and metabolic dysfunction (Deuschle et al., 1997; van den Berg et al., 1995; Qian et al., 2012). Glucocorticoid circadian and ultradian rhythmicity follow preceding changes in circulating ACTH. As with other steroid hormones, stimulation of glucocorticoid secretion by ACTH requires *de novo* synthesis. These ACTH-dependent glucocorticoid secretory episodes *in vivo*, are associated with rapid transcription of genes encoding steroidogenic proteins in the *zona fasciculata* of the adrenal cortex (Sewer and Waterman, 2002; Spiga et al., 2011a,b; Liu et al., 2013; Spiga et al., 2017), including the rate-limiting steroidogenic acute regulatory (StAR) protein (Stocco and Clark, 1996). Although initiation of steroidogenesis following ACTH stimulation depends on rapid post-translational modifications of StAR (Arakane et al., 1997), this protein is short-lived (Artemenko et al., 2001) and transcriptional episodes are essential for maintaining adequate mRNA and protein levels for subsequent secretory episodes (Ferguson, 1963; Garren et al., 1965; Clark et al., 1997).

The mechanism of action of ACTH involves cAMP/PKA-dependent mechanisms (Cammas et al., 1995; Schimmer, 1972; Clark et al., 2001), and, to a lesser extent, the Mitogen-activated protein kinase (MAPK) pathway (Gyles et al., 2001; Lotfi et al., 2000; Rocha et al., 2003; Winnay and Hammer, 2006). This results in activation of transcription factors responsible for inducing *Star* transcription, including steroidogenic factor 1 (SF-1) and cAMP response element binding protein (CREB) (Sandhoff et al., 1998; Aesoy et al., 2002; Manna et al., 2003; Lefrancois-Martinez et al., 2011). The transcription factor coactivator, CREB-regulated transcription coactivator (CRTC, previously known as Transducer of Regulated CREB activity; TORC), has been shown to enhance CREB binding to the RNA polymerase II pre-initiation complex at the promoter, through its binding to the CREB bZIP domain (Conkright et al., 2003; Orphanides et al., 1996). Three isoforms of CRTC have been identified, CRTC1, CRTC2 and CRTC3, (Conkright et al., 2003; Iourgenko et al., 2003), with *Crtc1* mRNA being located predominantly in the brain, and *Crtc2* and *Crtc3* ubiquitously expressed (Conkright et al., 2003; Wu et al., 2006; Watts et al., 2011; Uebi et al., 2010). Whilst in basal conditions CRTC remains sequestered in the cytoplasm, activation by administration of cAMP or forskolin leads to dephosphorylation and subsequent translocation of CRTC into the nucleus (Bittinger et al., 2004; Lee et al., 2015; Takemori et al., 2007), where it is required for maximal CREB-mediated transcriptional activity (Conkright et al., 2003; Liu et al., 2010; Wang et al., 2010). CRTC2 has been implicated in the regulation of *Star* transcription in transfected adrenocortical cells (Lee et al., 2015; Takemori et al., 2007). *In vivo*, in the rat, both ACTH injection and stress rapidly (within 5–7 min) induce CRTC2 dephosphorylation and nuclear translocation in the adrenal *zona fasciculata*, and is followed by an increase in *Star* transcription by 15 min (Liu et al., 2013; Spiga et al., 2011a,b).

The above evidence strongly suggests that CRTC2 is involved in the regulation of *Star* transcription. The adrenal cortex also expresses CRTC1 and CRTC3, though levels of CRTC1 are much lower than those of CRTC2 and CRTC3 both in rat; Fig. S1, and in humans (Conkright et al., 2003). Thus, it is reasonable to hypothesize that, in addition to CRTC2 (Liu et al., 2013; Takemori et al., 2007; Spiga et al., 2011a,b), activation of the other isoforms, especially the highly expressed CRTC3, is also involved in the transcriptional regulation of *Star*. The objective of the present study was to elucidate the roles of different CRTC isoforms on ACTH-regulated *Star* transcription and the signalling pathways involved in this regulation. For this purpose, we investigated the dynamics of nuclear translocation of the three endogenous CRTC isoforms in response to ACTH, in relationship with the time course of *Star* transcription and CREB phosphorylation in two murine adrenal cell lines, Y1-BS1 (Watt and Schimmer, 1981), and ATC7-L (Ragazzon et al., 2006). In contrast with the original cell line, Y1 (Yasumura et al., 1966; Bloch and Cohen, 1960), widely used to study steroidogenesis (Whitehouse et al., 2002; Zaidi et al., 2014; Rainey et al., 2004), the sub-clone, Y1-BS1, is ACTH-responsive but as with the parent line, has the disadvantage of not producing corticosterone (Parker et al., 1985). The line ATC7-L, derived from an adrenal fasciculata tumour (induced by targeted expression of SV40 large T antigen (Sahut-Barnola et al., 2000)), produces corticosterone in response to physiological ACTH concentrations, as well as episodic secretion in response to ACTH pulses (Ragazzon et al., 2006; Hazell et al., 2019). Using these two ACTH-responsive cell lines, we examined the physiological role of CRTC2 and CRTC3 on *Star* transcription, employing siRNA knockdown, and chromatin immunoprecipitation assays to investigate the recruitment of CRTCs by the *Star* promoter.

## 2 Materials and methods

All chemicals were purchased from Sigma Aldrich unless otherwise stated.

### 2.1 Cell cultures, transfections and treatments

Mouse adrenocortical Y1-BS1 cells (kindly provided by Dr Bernard Schimmer, University of Toronto, ON), were cultured in MEMα (Gibco) containing 2.5% heat-inactivated foetal bovine serum (Gibco), 15% heat-inactivated horse serum (Gibco), 1% penicillin/streptomycin (pen/strep). Mouse adrenocortical ATC7-L cells were cultured in 0.005% poly-L-lysine (P1399) pre-coated flasks, in DMEM/F12-GlutaMAX medium (Gibco) containing 2.5% heat-inactivated horse serum, 2.5% heat inactivated fetal bovine serum, 1% pen/strep and 1% insulin, transferrin and sodium selenite (ITS; Gibco) (Ragazzon et al., 2006). Both cell lines were cultured at 37 °C under a 5% CO_2_-95% air atmosphere.

For CRTC silencing experiments, Y1-BS1 cells were transfected by electroporation (Nucleofector II, Amaxa, Lonza, Gaithersburg, MD, USA). Six million Y1-BS1 cells per cuvette in 100 μl solution V, were combined with 600 nM siRNA oligonucleotides and transfected using program L-033, 600 nM of scrambled control #1 siRNA (Thermofisher Scientific) for control groups; 300 nM *5*′*-*GGUCCUGGAUUUUUAGGGAtt*-3′ Crtc2* siRNA (Thermofisher Scientific) plus 300 nM scrambled control #1 siRNA for the CRTC2 knockdown group; 300 nM *5*′*-*GACCAAUUCUGAUUCUGCUtt*-3′ Crtc3* siRNA (Thermofisher Scientific) plus 300 nM scrambled control #1 siRNA for the CRTC3 knockdown group; 300 nM each, *Crtc2* and *Crtc3* siRNA for combined CRTC2 and CRTC3. Following transfection, cells were cultured for 48 h in supplemented MEMα prior to changing to supplement-free media containing 0.1% BSA for 1 h (Y1-BS1) or 24 h (ATC7-L cells) before experimentation. Cells were stimulated with either 10 nM synthetic ACTH (ACTH-(1–39)), 1 mM 8-Br-cAMP, 100 nM of phorbol 12-myristate 13-acetate (PMA) or 3 nM epidermal growth factor (EGF) for the times indicated. In experiments involving inhibitors, cells were pre-incubated for 15 min with 10 μM of the PKA inhibitor, H89, 1 μM the MEK1/MEK2 inhibitor, UO126 or 5 μM the calcineurin inhibitor, cyclosporine A (CsA), or vehicle (final concentration 0.5% DMSO).

### 2.2 Rat adrenal cell isolation

Female Sprague Dawley rats were decapitated following CO_2_ sedation, adrenal glands removed, decapsulated and quartered, and then digested with collagenase Type II, 2 mg/ml in DMEM/HEPES (Gibco), containing 1% pen/strep, 1% BSA fraction V, 0.02% Deoxyribonuclease I, for 20 min at 37 °C, 95% air/5%CO_2_ under agitation. Tissue was then sedimented, washed and resuspended in DMEM/HEPES containing 1% pen/strep, 1% BSA, 0.002% Deoxyribonuclease I and 0.01% Trypsin inhibitor, and mechanically dispersed by aspiration/release with a syringe attached to 3 mm tubing. The supernatant containing dispersed cells was filtered through a 100 μm nylon gauze, and the procedure repeated until the media became clear. A second collagenase incubation and dispersion was performed on undigested tissue. Pooled supernatant was centrifuged at 100×*g*, cell pellets resuspended and preincubated for 1 h in 20 ml DMEM/HEPES containing 0.1% BSA and 1% pen/strep, before resuspending in fresh medium at 250,000 cell/ml. Aliquots (1 ml) were incubated at 37 °C for the time periods and conditions indicated. Incubations were terminated by placing vials on ice, then cells pelleted by centrifugation for RNA isolation.

### 2.3 RNA isolation and RT-qPCR

Cells were harvested in TRIzol reagent (Thermofisher Scientific) and RNA extracted using phase separation with chloroform. Total RNA was purified from the aqueous phase using RNeasy mini kit reagents and column DNase digestion (Qiagen, Valencia, CA, USA), per manufacturer instructions. Complementary (c)DNA was reverse transcribed from 1000 ng of RNA as previously described (Liu et al., 2008). qPCR primers for murine *Star* primary transcript (prior to splicing to mRNA), or heteronuclear RNA (hnRNA), were designed spanning an intronic-exonic region: *Forward 5*′*-*TGTCTCGCTCGGGGTCACACA*-3′, Reverse 5*′*-*AGGCAGGGGCACCTCAAGCT*-3*′*,* Invitrogen). PCR reactions were performed using power SYBR green PCR mix (ThermoFisher Scientific), 166 nM of each primer and 2 μl cDNA, final volume 15 μl, in a spectrofluorometric thermal cycler 7900 HT Fast real-time PCR system (ThermoFisher Scientific) as previously described (Liu et al., 2010). Briefly, samples underwent denaturation at 50 °C for 2 min, 95 °C for 10 min, followed by 45 cycles at 95 °C for 15 s and 60 °C for 1 min hnRNA levels were calculated using relative quantification by standard curve, normalised to glyceraldehyde 3-phosphate dehydrogenase (GAPDH) mRNA (murine *Gapdh* mRNA: *Forward 5*′-CCATCACTGCCACCCAGAAGA*-3′, Reverse 5*′*-*GACACATTGGGGGTAGGAACA*-3*′*,* Invitrogen), as determined in separate qRT-PCR reactions. Absence of detection when omitting the reverse transcription enzyme Superscript III (Invitrogen) indicated a lack of genomic DNA contamination.

### 2.4 Western immunoblot analysis

Following treatment, cells were washed and collected in ice-cold PBS containing protease and phosphatase inhibitors (Pierce, Rockford, IL, USA). Nuclear and cytosolic proteins were extracted using the NE-PER Nuclear and Cytoplasmic Extraction Reagent kit (Pierce), whole cell protein extracts were obtained using Tissue Protein Extraction Reagent (T-PER; Pierce), per manufacturer instructions. Protein samples (18 μg) were separated in Tris-Glycine gels, transferred to PVDF membranes and incubated overnight at 4 °C with the following primary antibodies: rabbit anti-CRTC1 (1:1000 dilution; Cell Signalling, Danvers, MA, USA), rabbit anti-CRTC2 (1:2000; ST1099 EMD Millipore Billerica, MA, USA), rabbit anti-CRTC3 (1:1000; Cell Signalling), mouse anti-phospho-CREB (1:500; 10E9 EMD Millipore), goat anti-HDAC1 (1:1000; C-19, Santa Cruz Biotech., Dallas, TX, USA), goat anti-β-actin (1:1000; I-19, Santa Cruz Biotech.) or goat anti-vinculin (1:5000; N19, Santa Cruz Biotech). Membranes were washed and incubated for 1 h at room temperature with a horseradish peroxidase-conjugated secondary antibody at 1:10000 (donkey anti-rabbit) or 1:5000 (donkey anti-goat and goat anti-mouse) dilution. Detection of immunoreactive bands was performed using ECL Plus TM reagents (GE Amersham Biosciences, Pittsburgh, PA, USA) followed by exposure to BioMax MR film (Eastman Kodak, Rochester, NY, USA). Band intensity was semi-quantified using ImageJ (freely available https://imagej.nih.gov/ij/download.html). Results are expressed as fold-change over the control values after correction for protein loading using HDAC1 for the nucleus, β-actin or Vinculin for cytoplasm and whole cell.

### 2.5 Chromatin immunoprecipitation assay

Following ACTH treatment, ATC7-L (1.7 × 10^6^) cells were fixed with 1% formaldehyde, quenched with glycine (0.125M final concentration), washed with PBS, collected into tubes and pelleted by centrifugation. For pCREB and CRTC2 immunoprecipitation, chromatin was sheared by sonication, adapted from work described previously (Evans et al., 2013). Briefly, cell pellets were resuspended in Lysis buffer (0.5% SDS, Invitrogen; 1% Triton x-100; 10 mM KCl; 1.5 mM MgCl_2_; 1 mM EDTA pH 7.4, Ambion; 20 mM HEPES pH 7.3, ThermoFisher Scientific) with protease/phosphatase inhibitors, and incubated on ice before Dounce homogenisation (Kontes, Kimble Chase, Vineland, NJ, USA). Following centrifugation, nuclear pellets were resuspended in Sonication buffer (0.2% SDS, 0.1M KCl, 1.5 mM MgCl_2_, 1 mM EDTA, 20 mM HEPES pH 7.3) with protease/phosphatase inhibitors, and then sonicated by Bioruptor (Diagenode, Liège, Belgium) to generate 0.25–1 kb chromatin fragments. In a different set of experiments for CRTC3 immunoprecipitation, conducted at the University of Bristol laboratory, chromatin fragmentation was achieved using micrococcal nuclease (MNase), as described previously (Stubbs et al., 2018).

Immunoprecipitation was performed as previously described (Grontved et al., 2013; Grontved et al., 2010). DNA concentration was determined by absorbance at 260 nm and samples were diluted in ChIP dilution buffer (0.01% SDS; 1.1% Triton x-100; 1.2 mM 0.5M EDTA; 17 mM Tris-HCl pH 8.1; 170 mM NaCl) at equal DNA amounts (max 0.1% SDS), then pre-cleared by incubation with Protein A/G plus agarose beads (Santa Cruz Biotech). Protein G Magnetic beads (Active Motif, Carlsbad, CA, USA) were linked to either anti-CRTC2 antibody (1.2 μg; Bethyl, Mongomery, TX, USA), a 1:1 cocktail of anti-pCREB antibodies (6 μg; 06–519 and ChIPAb+, Millipore) (Liu et al., 2011), anti-CRTC3 antibody (2 μg; ab91654, AbCam, Cambridgeshire, UK) (Jurek et al., 2015), or ChIP-grade non-specific rabbit IgG (4 μg; Cell Signalling) in Low Salt buffer (0.1% SDS; 1% Triton x-100; 2 mM EDTA; 20 mM Tris HCl pH8.1; 150 mM NaCl). Pre-cleared chromatin (28–43 μg PCREB/CRTC2, 12–25 μg CRTC3) was incubated with antibody-linked beads (50 μl beads/ml chromatin) overnight at 4 °C, then washed in Low Salt buffer, High Salt buffer (0.1% SDS; 1% Triton x-100; 2 mM EDTA; 20 mM Tris HCl pH8.1; 500 mM NaCl), LiCl buffer (0.25M LiCl; 1% IGEPAL; 1% sodium deoxycholate; 1 mM EDTA; 10 mM Tris-HCl pH8.1) and Tris-EDTA (TE) buffer (10 mM Tris-HCl, 1 mM EDTA pH8.1). Samples were eluted in Elution buffer (10 mM Tris-HCl pH 8.0, 300 mM NaCl, 55 mM EDTA, 0.5% SDS) with Proteinase K at 65 °C overnight, then chromatin resuspended in water following phenol-chloroform-isoamyl alcohol extraction. Samples were stored at −80 °C prior to DNA quantification by qRT-PCR, as detailed above. Primers spanning the three putative CRE sites at −104 bp to −5 bp of the *Star* promoter (*Forward 5*′*-*TTCCATCCTTGACCCTCTGC*-3*′*, Reverse 5*′*-*AGATCAAGTGCGCTGCCTTA*-3*′, Invitrogen) were designed using NCBI Primer-BLAST, as determined via NCBI GenBank. Promoter pulldown was quantified using mouse genomic DNA, normalised to the total levels in the chromatin input (promoter content in chromatin not subjected to immunoprecipitation).

### 2.6 Statistical analyses

All data are expressed as mean ± SEM of values obtained from a minimum of three independent experiments. Normal distribution of the data and homogeneity of variance were verified using the Shapiro-Wilk test and Normal Q-Q plots, and the LEVENE test, respectively. Data were analysed using one-way ANOVA, Welch ANOVA or two-way ANOVA, as indicated in the figure legend. When appropriate, one- or two-way ANOVA was followed by Fisher Least Significant Difference (Fisher LSD) post-hoc test, Welch ANOVA was followed by the Dunnett T3 post-hoc test. Statistical significance was set at *P* ≤ 0.05, with a trend defined as *P* ≤ 0.1.

## 3 Results

### 3.1 Time course of the effect of ACTH on *Star* transcription, CREB phosphorylation and CRTC translocation in Y1-BS1 and ATC7-L cells

**Fig. 1:**
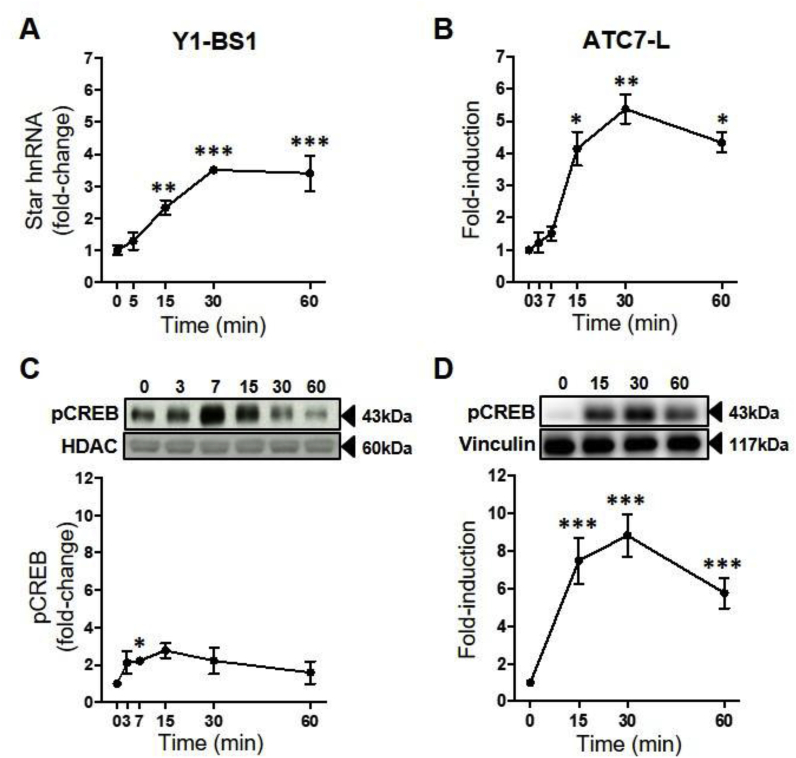
**Time course of the effect of ACTH on *Star* hnRNA and pCREB in Y1-BS1 and ATC7-L cells.** Y1-BS1 (**A** and **C**) and ATC7-L (**B** and **D**) cells were treated with 10 nM ACTH. Data points represent the mean ± SEM of *Star* hnRNA (**A-B**) and pCREB (**C-D**). *Star* hnRNA was normalised to *Gapdh* mRNA and is expressed as fold-changes of time 0 (n = 3–6/time point). In **C**, pCREB was measured in the nuclear extract, was normalised to HDAC, and is expressed as fold-changes of time 0 (n = 4–5/time point). In **D**, pCREB was measured in the whole cell extract, was normalised to vinculin, and is expressed as fold-change of time 0 (n = 3/time point). Data in **A** were analysed using one-way ANOVA followed by Fisher LSD post-hoc test; data in **B**, **C** and **D** were analysed using Welch ANOVA followed by Dunnett T3 post-hoc test. *P ≤ 0.05, **P ≤ 0.01, ***P ≤ 0.001 *vs* time 0.

The dynamics of *Star* transcription in response to ACTH were determined by measuring the levels of *Star* primary transcript (*Star* hnRNA). ACTH induced an overall increase in *Star* hnRNA levels both in Y1-BS1 and ATC7-L cells (*P* < 0.001; Fig. 1A and B), with no changes detected in the first 5–7 min, whereas a significant increase was observed at 15–60 min. Concomitant with the increase in *Star* hnRNA, ANOVA analysis revealed a significant effect of ACTH on pCREB in both cell lines (Y1-BS1: *P* = 0.02; Fig. 1C ATC7-L: *P* = 0.002; Fig. 1D). In Y1-BS1 cells, nuclear pCREB transiently increased 2.2-fold by 7 min, peaking at 15 min. In ATC7-L cells, pCREB increased by 7.5-fold at 15min and remained significantly elevated at 60 min (5.8-fold).

**Fig. 2:**
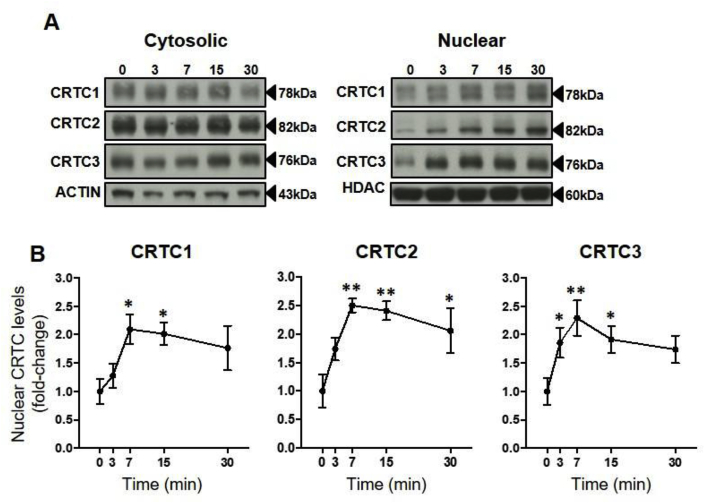
**Time course of the effects of ACTH on nuclear levels of CRTC in Y1-BS1 cells.** Cells were treated with 10 nM ACTH. (**A**) Representative Western immunoblot of cytoplasmic and nuclear CRTC1, CRTC2 and CRTC3 levels. (**B**) Semi-quantification of Western immunoblot data expressed as fold-change of time 0. Data are the mean ± SEM of nuclear levels of CRTC1, CRTC2, and CRTC3, normalised to HDAC (n = 4/time point). Data were analysed using one-way ANOVA followed by Fisher LSD post-hoc test. *P ≤ 0.05, **P ≤ 0.01, *vs* time 0.

**Fig. 3:**
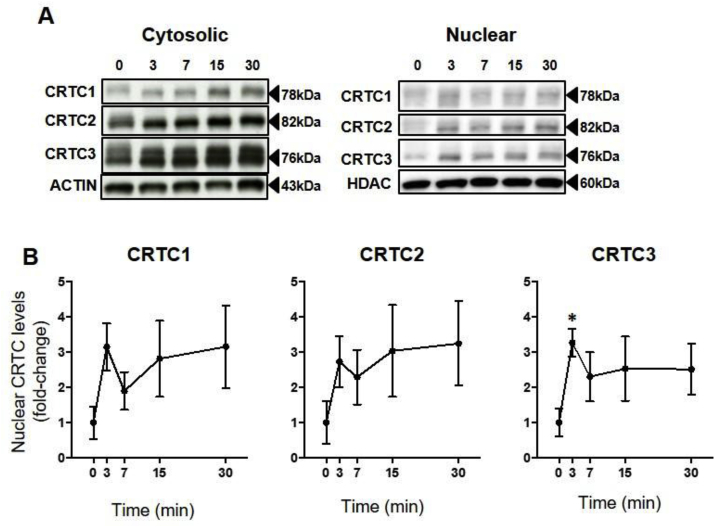
**Time course of the effects of ACTH on nuclear levels of CRTC in ATC7-L cells.** Cells were treated with 10 nM ACTH. (**A**) Representative Western immunoblot of cytoplasmic and nuclear CRTC1, CRTC2 and CRTC3 levels. (**B**) Semi-quantification of Western immunoblot data expressed as fold-change of time 0. Data are the mean ± SEM of nuclear levels of CRTC1, CRTC2, and CRTC3, normalised to HDAC (n = 4/time point). Data were analysed using Welch ANOVA followed by Dunnett T3 post-hoc test. *P ≤ 0.05 *vs* time 0.

To investigate whether these changes in pCREB and *Star* transcription are associated with differential changes in CRTC subtype nuclear translocation, we measured the dynamics of cytosolic and nuclear levels of CRTC1, CRTC2 and CRTC3. ACTH had no significant effect on cytosolic CRTC1, CRTC2 or CRTC3 levels in Y1-BS1 (Fig. 2A; data not shown) or ATC7-L cells (Fig. 3A; data not shown). Y1-BS1 cells exhibited a significant overall increase in nuclear translocation of CRTC1 (*P* = 0.04), CRTC2 (*P* = 0.01) and CRTC3 (*P* = 0.04; Fig. 2A and B). Nuclear levels of CRTC3 were already significantly increased by 3 min, whilst nuclear levels of CRTC1 and CRTC2 were significantly increase at 7 min. A significant overall effect of ACTH on nuclear CRTC1 and CRTC3 was also detected in ATC7-L cells (CRTC1: *P=*0.05; CRTC3: *P* = 0.02; Fig. 3A and B), with nuclear levels of CRTC3 also significantly increased by 3 min. Despite an increase in nuclear levels of CRTC2 in each one of four experiments (range 1.5–5.3-fold at 3 min), the changes were not significant due to high variability in the magnitude of the effect across experiments (*P* = 0.22).

### 3.2 ACTH induces rapid association of pCREB, CRTC2 and CRTC3 at the proximal *Star* promoter in ATC7-L cells

**Fig. 4:**
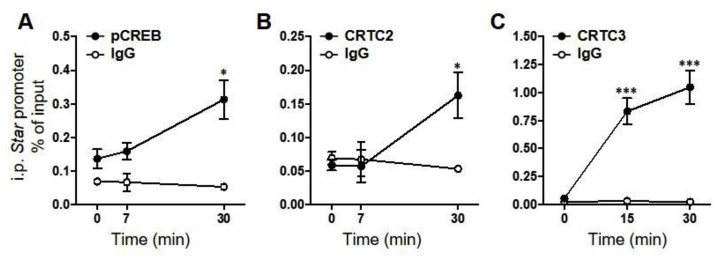
**ACTH-induced binding of pCREB, CRTC2 and CRTC3 to the *Star* promoter in ATC7-L cells.** Cells were treated with 10 nM ACTH prior to chromatin immunoprecipitation by antibodies for pCREB (**A**), CRTC2 (**B**) and CRTC3 (**C**) proteins and normal rabbit IgG. Binding of each protein target to the *Star* promoter was calculated as percentage pulldown of the input. Data represent the mean ± SEM of data obtained in 3–4 independent experiments. pCREB, CRTC2 and CRTC3 data were analysed using one-way ANOVA followed by Fisher LSD post-hoc test. IgG data were analysed using Welch ANOVA (**A-B**) and one-way ANOVA (**C**). *P ≤ 0.05, ***P < 0.001 *vs* 0 min.

The increases in nuclear CRTCs, preceding increases in *Star* hnRNA, suggest an involvement of the coactivators in the initiation of *Star* transcription. To determine whether CRTC2 and CRTC3 can interact with the *Star* promoter, we used the adrenocortical cell line, ATC7-L, and ChIP assays to measure recruitment of pCREB and CRTC proteins by the *Star* promoter. Recruitment of CRTC1 could not be measured because of the lack of a suitable antibody for ChIP. ACTH significantly increased the binding of pCREB (*P* = 0.04; Fig. 4A) and CRTC2 (*P* = 0.04; Fig. 4B) at the *Star* promoter. No increases in binding were detected at 7 min, but by 30 min, there was a significant increase above basal levels for both pCREB (*P* = 0.02) and CRTC2 (*P* = 0.02). Immunoprecipitation of the *Star* promoter by the CRTC3 antibody also revealed a significant effect of ACTH on CRTC3 binding (*P* < 0.001; Fig. 4C). Binding of CRTC3 to the *Star* promoter was detected by 15 min (*P* = 0.001), the earlier point measured, and remained at similar levels at 30 min (*P* < 0.001).

### 3.3 Knockdown of CRTC2 and CRTC3 attenuates *Star* transcription

**Fig. 5:**
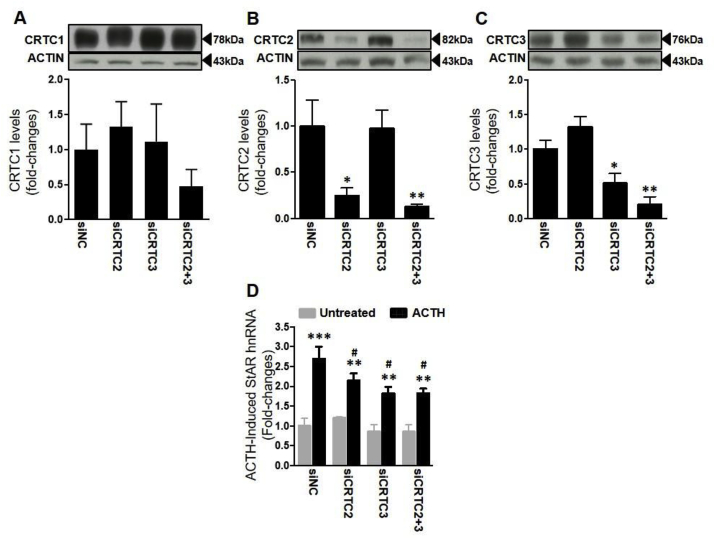
**Effect of CRTC2 and CRTC3 silencing on ACTH-induced increases in *Star* hnRNA levels in Y1-BS1 cells.** Y1-BS1 cells were transfected with non-coding siRNA (siNC) or siRNA oligonucleotides for CRTC2 (siCRTC2), CRTC3 (siCRTC3), or both (siCRTC2+3), 48 h prior to treatment with 10 nM ACTH for 45min. (**A, B** and **C**) Efficacy of silencing determined by Western immunoblot and semi-quantification of the levels of whole cell CRTC1, CRTC2 and CRTC3, normalised to actin, expressed as fold-change levels of the siNC transfected control group. No effect on CRTC1 levels was observed following transfection of siCRTC1 oligonucleotides (data not shown). (**D**) Effect of CRTC siRNAs on *Star* hnRNA levels, normalised to *Gapdh* mRNA and expressed as fold-change of siNC transfected controls. Data are the mean ± SEM of data obtained in 3 independent experiments. Data in **A, B** and **C** were analysed using one-way ANOVA followed by Fisher LSD post-hoc test; data in **D** were analysed using two-way ANOVA followed by Fisher LSD post-hoc test. *P ≤ 0.05, **P ≤ 0.01, ***P ≤ 0.001 *vs* untreated control. ^#^*P* ≤ 0.05 *vs* ACTH-treated siNC control.

The involvement of the different CRTC subtypes on ACTH-induced *Star* transcription was investigated further using siRNA oligonucleotides to inhibit the expression of CRTC1-3 in the adrenocortical cell line, Y1-BS1. While technical difficulties impaired CRTC1 knockdown, transfection with *Crtc2* and *Crtc3* siRNA oligonucleotides, alone or in combination effectively reduced the respective CRTC in whole cell protein extracts, for CRTC2 (*P* = 0.01; Fig. 5B) and CRTC3 (*P* = 0.002; Fig. 5C) protein levels. Transfection with *Crtc2* siRNA decreased CRTC2 protein by 75.3% ± 8.4 (*P* = 0.02 vs non-coding siRNA sequence, siNC; Fig. 5B), whilst Crtc3 siRNA transfection decreased CRTC3 protein by 49.2% ± 14.3 (*P* = 0.03 vs siNC; Fig. 5C). Furthermore, there was no significant difference in whole cell CRTC2 and CRTC3 protein levels when cells were transfected with the heterologous siRNA, confirming specificity of the siRNA used. Knockdown of CRTC2 and CRTC3, alone or combined, did not affect CRTC1 protein levels (*P* = 0.49; Fig. 5A).

*Star* hnRNA levels were then measured either following 45 min treatment with 10 nM ACTH or vehicle (Fig. 5D). Two-way ANOVA revealed an overall significant effect for both ACTH and siRNA on *Star* hnRNA (*P* < 0.001 and P = 0.03, respectively), but no interaction (*P* = 0.15). ACTH increased *Star* hnRNA levels in siNC cells (*P* < 0.001), an effect which was significantly attenuated in cells transfected with either *Crtc2* or *Crtc3* siRNA, or their combination. Although ACTH-stimulated values were still significantly higher than the respective basal (*Crtc2* siRNA: *P* = 0.002; *Crtc3* siRNA: P = 0.002; Crtc2+Crtc3 siRNA: P = 0.002), responses to ACTH were significantly lower than in siNC (Crtc2 siRNA: *P* = 0.05; *Crtc3* siRNA: *P* = 0.004). No additivity was observed between the inhibitory effect of *Crtc2* and *Crtc3* siRNAs on ACTH-stimulated Star hnRNA, with the combined effect being similar to that of the individual oligonucleotides (*P* = 0.004, compared to siNC cells). No significant differences in basal *Star* hnRNA levels between siRNA treatment groups were found.

### 3.4 Stimulation of cAMP, but not of MAPK or PKC pathways, mimics the effect of ACTH on *Star* transcription

**Fig. 6:**
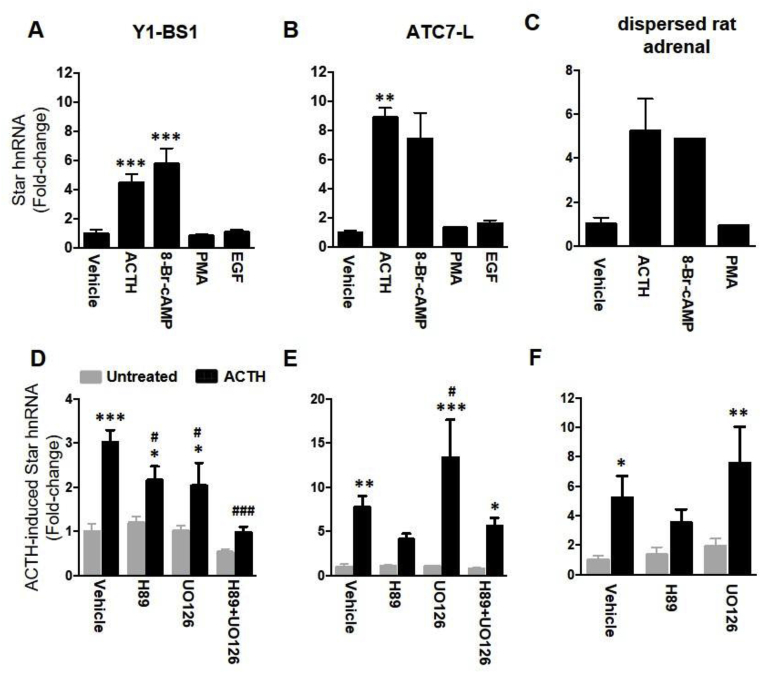
**Effect of mimicry and inhibition of ACTH signalling on Star hnRNA levels in Y1-BS1, ATC7-L and dispersed rat adrenals cells.** Star hnRNA levels were measured either prior to or 30 min after treatment with 10 nM ACTH, 1 mM 8-Br-cAMP, 100 nM PMA or 3 nM EGF in Y1-BS1 (**A**) and ATC7-L (**B**), or 60 min after treatment in dispersed rat adrenal cells (**C**). For inhibition studies, Y1-BS1 cells (**D**), ATC7-L (**E**) and dispersed rat adrenal cells (**F**) were pre-incubated for 15 min with either vehicle (0.5% DMSO), 10 μM H89, 1 μM UO126, or 10 μM H89+1 μM UO126 before addition of ACTH at a final concentration of 10 nM. Bars represent the mean ± SEM of 3–6 experiments, normalised to *Gapdh* mRNA and expressed as fold-change from basal vehicle values. PMA treated cells in **C** represent the mean of data from 2 independent experiments. Data in **A** were analysed using one-way ANOVA followed by Fisher LSD post-hoc test; data in **B** were analysed using Welch ANOVA followed by Dunnett T3 post-hoc test. Data in **D**, **E** and **F** were analysed using Two-way ANOVA followed by Fisher LSD post-hoc test. *P ≤ 0.05, **P ≤ 0.01, ***P ≤ 0.001 vs untreated vehicle control. #P ≤ 0.05, ###P ≤ 0.001 vs ACTH-treated vehicle control.

To test whether other signalling pathways, in addition to ACTH/cAMP, can regulate *Star* transcription, Y1-BS1 and ATC7-L were treated for 30 min with either ACTH, 8-Br-cAMP, the PKC stimulator PMA, or the MAPK stimulator EGF (Fig. 6A and B). In Y1-BS1 cells (Fig. 6A), both ACTH (*P* = 0.001) and 8-Br-cAMP (*P* < 0.001) significantly increased the levels of *Star* hnRNA, whilst treatment with PMA or EGF had no effect on *Star* hnRNA levels. In ATC7-L cells (Fig. 6B), *Star* hnRNA levels also increased in cells treated with ACTH (*P* = 0.007). In each of the three experiments, 8-Br-cAMP increased in *Star* hnRNA levels, but due to variability in the magnitude of the increase (range 5.4–11.0-fold), the effect was not statistically significant (*P* = 0.23). Consistent with the findings in Y1-BS1 cells, neither PMA nor EGF had any effect on *Star* hnRNA levels in ATC7-L cells. These findings were confirmed in dispersed rat adrenal cells, with both ACTH and 8-Br-cAMP increasing *Star* hnRNA levels, while no effect of PMA treatment was seen (Fig. 6C).

### 3.5 ACTH-stimulated *Star* transcription is attenuated by inhibition of PKA and MAPK activity in a cell line specific manner

To further study the signalling pathways mediating *Star* transcription, murine adrenocortical cell lines were pre-treated with either vehicle, the PKA inhibitor H89, the MEK inhibitor UO126, or with a combination of both inhibitors for 15 min prior to incubation with 10 nM ACTH for 30 min**.** The inhibitors, alone or in combination, had no significant effect on basal *Star* hnRNA levels (Fig. 6D–F). Both Y1-BS1 (Fig. 6D) and ATC7-L (Fig. 6E) cells showed a significant effect of ACTH (*P* < 0.001), inhibitors pre-treatment (Y1-BS1: *P* < 0.001; ATC7-L: *P* = 0.03), and a significant interaction (Y1-BS1: *P* = 0.01; ATC7-L: *P* = 0.03).

When compared with the respective basal, ACTH treatment in Y1-BS1 cells increased *Star* hnRNA levels in cells pre-treated with vehicle (*P* < 0.001), UO126 (*P* = 0.03) or H89 (*P* = 0.01), but the effect of ACTH was significantly attenuated in cells pre-treated with H89 (*P* = 0.02) and UO126 (*P* = 0.01) compared vehicle pre-treatment. Furthermore, pre-treatment with the combination of H89 and UO126 had a significant additive inhibitory effect (*P* < 0.001), preventing a significant stimulatory effect of ACTH (*P* = 0.26 vs basal). Similarly, ACTH increased *Star* hnRNA levels in ATC7-L cells pre-treated with vehicle (*P* = 0.004), UO126 (*P* < 0.001) and H89 + UO126 (*P* = 0.01) compared with respective basal values, but pre-treatment with H89 alone only tended to reduce the stimulatory effect of ACTH (*P=*0.15). Compared with ACTH treatment in vehicle pre-treated controls, ACTH-stimulated *Star* hnRNA levels also tended to be lower in H89 pre-treated cells (*P* = 0.09). The lack of a significant effect of H89 in the overall analysis was likely due to the wide range in the magnitude of the ACTH responses in the UO126 group, since there was a significant reduction when comparing ACTH-stimulated values in H89 and vehicle pre-treated groups by *t*-test analysis (*P* = 0.04). In contrast to Y1-BS1 cells, preincubation of ATC7-L cells with the MAPK inhibitor, UO126, significantly augmented the stimulatory effect of ACTH on *Star* hnRNA levels compared with ACTH in vehicle pre-treated cells (*P* = 0.02).

Similar to the effects in the cell lines, in vehicle pre-treated collagenase-dispersed rat adrenal cells (Fig. 6F) there was a significant effect of ACTH (*P* = 0.002), while the overall effect of the inhibitors or their interaction was not significant (*P* = 0.213 and *P* = 0.412, for inhibitors and interaction, respectively). As in ATC7-L cells, *Star* hnRNA increases in the ACTH-treated vehicle (*P* = 0.035) and UO126 (*P* = 0.008) groups were significant compared to basal, whilst pre-incubation with H89, but not with UO126, blunted S*tar* hnRNA response to ACTH (*P* = 0.242). Also similar to ATC7-L cells, pre-incubation with UO126 did not inhibit but tended to increase the effect of ACTH compared with vehicle pre-treated cells.

### 3.6 Inhibition of PKA and calcineurin decreases ACTH-mediated nuclear translocation of CRTC1, CRTC2 and CRTC3 in ATC7-L cells but not ACTH-mediated *Star* transcription

**Fig. 7:**
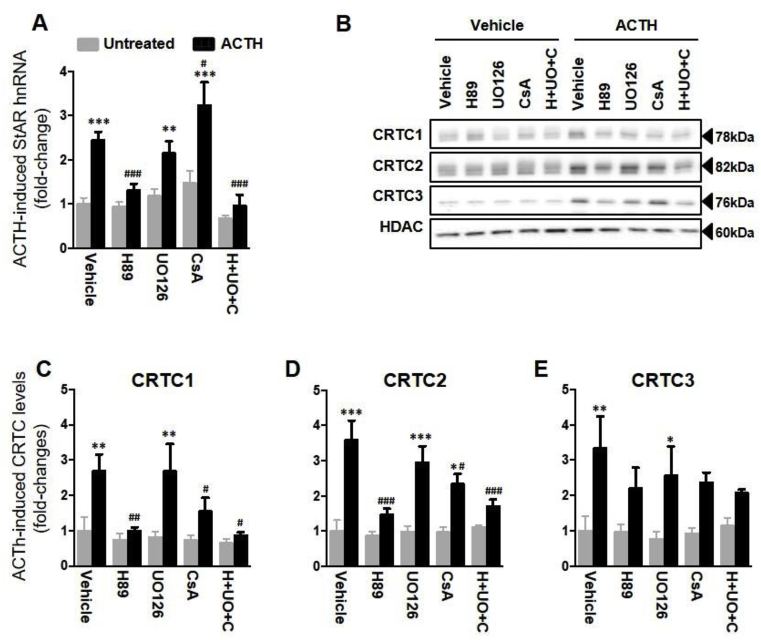
**Effect of inhibition of ACTH signalling on *Star* hnRNA levels and CRTC activity in ATC7-L cells.** Cells were pre-treated for 15 min with either vehicle (0.5%DMSO), protein kinase A inhibitor H89 (H, 10 μM), the MAP kinase inhibitor UO126 (U, 1 μM) or the calcineurin inhibitor CsA (5 μM), either alone or in combination (H89 + UO126 + CsA), prior to treatment with 10 nM ACTH for 10 min. (**A**) RT-qPCR quantification of *Star* hnRNA, normalised to *Gapdh* mRNA and expressed as fold-change levels of the basal vehicle control. Bars represent the mean ± SEM of 3–5 independent experiments. (**B**) Representative Western immunoblot of nuclear CRTC1, CRTC2 and CRTC3 levels. Quantification of Western immunoblot data of CRTC1 (**C**), CRTC2 (**D**), and CRTC3 (**E**); data are normalised to HDAC and expressed as fold-change of basal. Bars represent the mean ± SEM of 3–5 independent experiments. Data were analysed using two-way ANOVA followed by Fisher LSD post-hoc test. *P ≤ 0.05, **P ≤ 0.01, ***P ≤ 0.001 *vs* untreated vehicle control. ^#^*P* ≤ 0.05, ^##^*P* ≤ 0.01, ^###^*P* ≤ 0.001 *vs* ACTH-treated vehicle control.

To investigate the role of PKA, MEK and calcineurin on CRTC-mediated regulation of ACTH-induced *Star* transcription, the effect of 10 min treatment with ACTH on *Star* hnRNA levels and nuclear levels of CRTC1, CRTC2 and CRTC3 were measured in ATC7-L cells pre-treated with UO126, H89 or the calcineurin inhibitor CsA. There was a significant effect of ACTH (*P* < 0.001) and inhibitors treatment (*P* < 0.001), as well as interaction (*P* = 0.006), on *Star* hnRNA levels (Fig. 7A). ACTH significantly increased *Star* hnRNA in cells pre-incubated with vehicle (*P* < 0.001), UO126 (*P* = 0.003) and CsA (*P* < 0.001) but not in cells pre-incubated with H89 alone (*P* = 0.17) or in combination with the other antagonists (H89 + UO126 + CsA: *P* = 0.37). Interestingly, the effect of ACTH on *Star* hnRNA was potentiated by CsA (*P* = 0.01 vs ACTH alone), whereas the potentiation by UO126 observed after 30 min ACTH treatment (Fig. 6D) was not present after 10 min treatment.

Treatment with ACTH for 10 min also exerted a significant overall effect on nuclear levels of CRTC1 (*P* = 0.001; Fig. 7B and C), CRTC2 (*P* < 0.001; Fig. 7B and D) and CRTC3 (*P* < 0.001; Fig. 7B and E). Furthermore, the effect of inhibitors pre-treatment was significant for nuclear CRTC1 (*P* = 0.04; interaction *P* = 0.09) and CRTC2 (*P* = 0.006; interaction *P* = 0.011), but not for nuclear CRTC3 (*P* = 0.76; interaction *P* = 0.74). ACTH significantly increased nuclear levels of CRTC1, CRTC2 and CRTC3 in cells pre-treated with vehicle (CRTC1: *P* = 0.001; CRTC2: *P* < 0.001; CRTC3: *P* = 0.002) and UO126 (CRTC1: *P* = 0.002; CRTC2: *P* < 0.001; CRTC3: *P* = 0.03), whilst nCRTC2 levels alone were also significantly increased in CsA pre-treated cells (*P* = 0.016). Furthermore, ACTH-induced levels of nuclear CRTC1 and CRTC2 were significantly reduced in cells treated with the inhibitors H89 (CRTC1: *P* = 0.02; CRTC2: *P* < 0.001), CsA (CRTC1: *P* = 0.05; CRTC2: *P* = 0.01) and the combined H89 + UO126 + CsA (CRTC1: *P* = 0.003; CRTC2: *P* < 0.001), when compared to cells pre-treated with vehicle. However, H89 and CsA only tended to attenuate the effect of ACTH on nuclear levels of CRTC3 (H89: *P* = 0.09; CsA: *P* = 0.13, compared with ACTH stimulation in vehicle treated cells). Furthermore, the effect of pre-treatment all 3 combined inhibitors on ACTH-stimulated nuclear accumulation of CRTC3 was no different from the effects of each single inhibitor (*P* = 0.31 vs basal).

## 4 Discussion

This *in vitro* study shows that nuclear translocation of the 3 isoforms of endogenous CRTC (CRTC1, CRTC2 and CRTC3) parallels or precedes the increases in *Star* hnRNA induced by ACTH, in agreement with previous reports of *ex vivo* work in rats. Using chromatin immunoprecipitation and siRNA knockout we now demonstrate that CRTC2 and CRTC3 are involved in the regulation of *Star* transcription by ACTH in a PKA signalling dependent manner. The use of ACTH-responsive adrenocortical Y1-BS1 and ATC7-L cell lines allowed us to study the effect of physiological stimulator, ACTH, on endogenous proteins, rather than cAMP analogues, as with most previous studies (Takemori et al., 2007; Whitehouse et al., 2002; Zaidi et al., 2014; Jefcoate et al., 2011; Manna et al., 2002). The effects of ACTH on *Star* transcription and on nuclear pCREB and CRTC were similar in both cell lines, however, the actions of signalling inhibitors differed, with the MEK inhibitor, UO126, having an inhibitory effect in Y1-BS1 cells and a potentiating effect in ATC7-L cells. Interestingly, the effect of signalling inhibitors in ATC-7 cells resembles that observed in collagenase dispersed rat adrenal cells, suggesting that, at least in rodents, they are more representative of normal adrenal fasciculata cells. However, resistance to transfection made ATC7-L cells unsuitable for use in CRTC knock down experiments.

Whilst in earlier studies ACTH increases *Star* mRNA levels within 2 h in Y1-BS1 cells (Lin et al., 2001) and by 30 min in ATC7-L cells (Ragazzon et al., 2006), measuring *Star* hnRNA made it possible to show significant transcriptional activation in response to ACTH by 15 min in both cell lines. These rapid increases are consistent with findings in rat adrenal tissue 15 min after ACTH injection (Spiga et al., 2011a,b, 2017), or cAMP incubation in Y1 cells (Lee et al., 2015; Jefcoate et al., 2011). Consistent with the view that CREB phosphorylation is critical for *Star* transcription (Spiga et al., 2011a,b, 2017; Manna et al., 2003; Lefrancois-Martinez et al., 2011), ACTH treatment induced rapid and transient increases in nuclear pCREB in both cell lines.

Rapid ACTH-induced nuclear translocation of all 3 CRTC isoforms (CRTC1, 2 and 3) by 10 min in both Y1-BS1 cells and ATC7-L cells, coinciding with the earliest detected increases in *Star* hnRNA following ACTH exposure, supports a role for CRTC in the initiation of *Star* transcription. These kinetics differ from previous findings in transfected Y1 cells, showing nuclear translocation of CRTC2, but not CRTC1 or CRTC3, in response to cAMP stimulation (Lee et al., 2015). In keeping with the rapid nuclear translocation of the three CRTC isoforms shown here, using ATC7-L cells we demonstrate, for the first time, an early interaction of CRTC2 (by 30 min) and CRTC3 (by 15 min) with DNA fragments including the −104 bp to −5 bp *Star* promoter region containing three cAMP-response element (CRE) half-sites (Manna et al., 2002). The present finding of pCREB binding to the *Star* promoter at 30 min ACTH incubation is consistent with previous observations in Y1 cells stimulated with cAMP analogues (Jefcoate et al., 2011). The latter study also showed delayed (60 min) CRTC2 recruitment, from which authors concluded that CRTC2 is involved in maintaining, rather than initiating, high rates of *Star* transcription. The difference between the former (Lee et al., 2015; Jefcoate et al., 2011) and present findings could reflect the use of different cell lines, or studying the effect of ACTH on endogenous CRTC2 as opposed to the effect of cAMP on transfected protein (which may exhibit altered intracellular localisation and bioactivity). Interestingly, in contrast to the parallel nuclear translocation patterns of the three CRTC isoforms, extrapolation of the ChIP time courses suggests that ACTH-induced recruitment of CRTC3 by the *Star* promoter, which is already maximal at 15 min, could potentially precede that of CRTC2. Although CRTC2 recruitment was not measured at 15 min, the lack of any promoter association at 7 min renders it unlikely that binding is maximal at 15 min. Since CRTC lacks DNA-binding activity, its recruitment by the *Star* promoter CRE requires association with pCREB through its CREB binding domain (CBD) (Luo et al., 2012). Amino acid substitution studies have shown that small changes in key motifs of the CBD region of CRTC2 can impact its binding affinity with pCREB (Conkright et al., 2003; Luo et al., 2012). Thus, while the similar nuclear translocation time courses of CRTC1-3 could predict parallel dynamics of recruitment to the *Star* promoter, differences in pCREB affinity between the three CRTC isoforms could affect the recruitment time by the *Star* promoter. Testing this possibility will require further detailed analyses of the early kinetics of the three CRTC isoform pCREB association and recruitment by the *Star* promoter.

Previous reports demonstrate that CRTC2 overexpression increases *Star* transcription (Takemori et al., 2007), while activation of salt inducible kinase (SIK) 1, which prevents activation and nuclear localisation of CRTC2, inhibits *Star* transcription (Lin et al., 2001; Katoh et al., 2004). We demonstrate that siRNA knockdown of CRTC2 and CRTC3 in Y1-BS1 cells only partially inhibits ACTH stimulation of *Star* hnRNA levels, directly establishing a role for CRTC mediating ACTH-induced *Star* transcription. However, in contrast to the full inhibition of cAMP-dependent *Crh* transcription by simultaneous knockdown of CRTC2 and CRTC3 seen in rat hypothalamic 4B cells (Liu et al., 2010), knocking down both CRTC isoforms in Y1-BS1 cells had no additive effect on *Star* transcription after 45 min ACTH exposure. This suggests CRTC2 and CRTC3 both act through a similar mechanism of action, and that at the time of maximal Star transcription either CRTC2 of CRTC3 are capable of inducing full activation. However, it is possible that differential recruitment of CRTC isoforms by the atypical *Star* half-CREs (Luo et al., 2012; Song et al., 2018), due to differential affinity with pCREB or other mechanism (Conkright et al., 2003; Luo et al., 2012), could have different functional implications at earlier time points of ACTH stimulation.

It is also evident from these experiments that other factors are capable of mediating considerable ACTH-stimulation of Star transcription in the absence of both CRTC2 and CRTC3. Although CRTC1 expression is far lower than that of CRTC2 and CRTC3 in rat (Fig. S1) and human adrenals (Conkright et al., 2003), we show clear nuclear translocation of CRTC1 following ACTH treatment, suggesting a possible role for CRTC1 in *Star* transcription. This could not be examined in the present study because of the inability to knockdown CRTC1 protein in Y1-BS1 cells, and future studies will be needed for elucidating the potential role of CRTC1 on *Star* transcription. In addition, SF-1, which is upregulated by ACTH (Ragazzon et al., 2006; Hazell et al., 2019), binds the *Star* promoter and is necessary for cAMP-induced *Star* transcription (Sandhoff et al., 1998; Sugawara et al., 1997). Furthermore, subsets of CREB-inducible genes can be alternatively regulated by CREB coactivator CREB binding protein (CBP) (Kasper et al., 2010). The role of these factors and their interaction with the transcriptional complex during Star transcriptional initiation will require further investigation.

Investigation of signalling pathways showed that whilst MAPK activation by EGF had no effect on *Star* transcription in either Y1-BS1 or ATC7-L cells, MEK inhibition by UO126 attenuated ACTH-induced *Star* hnRNA in Y1-BS1 cells, but not ATC7-L cells. Furthermore, combination of H89 and UO126 abolished *Star* transcriptional responses to ACTH in Y1-BS1 cells, suggesting both pathways are required for full cAMP-dependent stimulation of *Star* transcription in this cell line. Similar results were found when using the PKA inhibitor PKI or MAPK inhibitor SL327 (Smith, Huang and Aguilera, unpublished observations). Previous studies also suggest cell-specific differences in the MAPK involvement in *Star* transcriptional regulation; Lefrancois-Martinez et al. (2011) found no effect of MAPK inhibition on ACTH-stimulated StAR protein levels in ATC1 cells, whilst, Gyles et al. (2001) showed inhibition of forskolin-stimulated *Star* mRNA and protein by MEK inhibitors in Y1 cells. Transactivation of the MAPK pathway by ACTH and cAMP has been implicated in steroidogenesis (Gyles et al., 2001; Winnay and Hammer, 2006). Furthermore, consistent with previous reports (Le and Schimmer, 2001), ACTH increased pERK levels in Y1-BS1 cells and, to a lesser extent, ATC7-L cells (Smith, Olah and Aguilera, unpublished observations).

H89 blunted ACTH-induced increases in nuclear CRTC, consistent with the established cAMP dependence of CRTC activation and translocation to the nucleus (Bittinger et al., 2004; Takemori et al., 2007; Liu et al., 2010). This was associated with complete inhibition of ACTH-induced *Star* hnRNA at 10 min, while there was a partial recovery by 30 min. This is consistent with the effects of siRNA CRTC knockdown, in which there was only a partial reduction of *Star* hnRNA by 45 min ACTH stimulation, despite reduced CRTC2 and CRTC3 levels. Although earlier *Star* hnRNA responses to ACTH were not measured in the siRNA experiments, the overall findings strongly suggest that PKA/CRTC mechanisms are essential for early transcriptional activation of *Star*, and that additional signalling mechanisms are important to sustain the activation. The fact that MAPK appears to play a role in CRTC regulation only in Y1-BS1 cells, raises a note a caution when extrapolating data obtained from cell lines to *in vivo* regulation in various species.

No effect of PMA indicates PKC activation alone is insufficient for initiating *Star* transcription. PKC has previously been implicated in the regulation of StAR protein levels in bovine adrenal cells (Nishikawa et al., 1996), whilst low levels of steroidogenesis were previously induced by 2 h incubation with PMA in Y1 cells (Frigeri and Armelin, 1996). Furthermore, Leydig M-10 cell studies have shown phorbol esters increase *Star* mRNA levels (Manna and Stocco, 2005; Manna et al., 2011). Ca^2+^-sensitive calcineurin can be activated by cAMP (Antoni, 1996), and it is known to activate CRTC (Bittinger et al., 2004; Lee et al., 2015; Rahnert et al., 2016; Screaton et al., 2004). Thus, ACTH signalling could lead to calcineurin activation and play a role in CRTC activation and nuclear translocation in the adrenal fasciculata. However, CsA potentiated the effect of ACTH on *Star* transcription, despite attenuating ACTH-induced nuclear translocation of CRTC2. It is noteworthy that Y1-BS1 cells lack part of the steroidogenic enzyme expression profile, which characterises *zona fasciculata* cells (Bloch and Cohen, 1960; Parker et al., 1985). The lost differentiation of these cells may be responsible for the apparent dependence of *Star* transcription on calcium (Lee et al., 2015) and MAPK signalling (Gyles et al., 2001), that was not observed in ATC7-L cells in this study or elsewhere (Lefrancois-Martinez et al., 2011; Schiebinger et al., 1985). Furthermore, CsA only partially inhibits CRTC1 and CRTC2 translocation, suggesting PKA may be the main mechanism regulating CRTC1 and CRTC2 activity. Although less sensitive to calcium signalling than *zona glomerulosa* cells (Braley et al., 1986; Omura et al., 2007; Spat et al., 2016), intracellular calcium depletion in rat *zona fasciculata* cells blunts cAMP-induced corticosterone release, suggesting that cAMP activation of adrenal steroidogenesis in the adrenal *fasciculata* requires calcium release from intracellular stores (Schiebinger et al., 1985).

This study provides evidence that of the 3 isoforms of CRTC present in the adrenal fasciculata, at least CRTC2 and CRTC3 are involved in the initiation of *Star* transcription. The rapid nuclear translocation and recruitment by the *Star* promoter of CRTC2 and CRTC3 in response to ACTH, preceding the maximal increases in *Star* primary transcript, and the ability of siRNA knockdown of these CRTC isoforms to attenuate ACTH-induced *Star* transcription, strongly suggest that PKA-mediated activation of CRTC2 and CRTC3 plays a role in the initiation of *Star* transcription. However, the fact that knockdown of both subtypes (or preventing their activation by PKA inhibitors) reduces but does not prevent ACTH-induced *Star* hnRNA increases, indicates the participation of additional factors and emphasizes the complexity of the mechanisms regulating StAR expression.

## Declaration of competing interest

Lorna Smith, Victoria Huang, Mark Olah, Loc Trinh, Ying Liu, Georgina Hazell, Becky Conway-Campbell, Zidong Zhao, Antoine Martinez, Anne-Marie Lefrançois-Martinez, Stafford Lightman, Francesca Spiga and Greti Aguilera declare no conflict of interest.
