# Supplementary material for: Involvement of CREB-regulated transcription coactivators (CRTC) in transcriptional activation of steroidogenic acute regulatory protein (Star) by ACTH
Source: Mol Cell Endocrinol. 2020 Jan 1;499:110612. doi: 10.1016/j.mce.2019.110612 (PMC6899503; doi:10.1016/j.mce.2019.110612)
Supplement: Multimedia component 2 [file mmc2.pptx]

## Slide 1
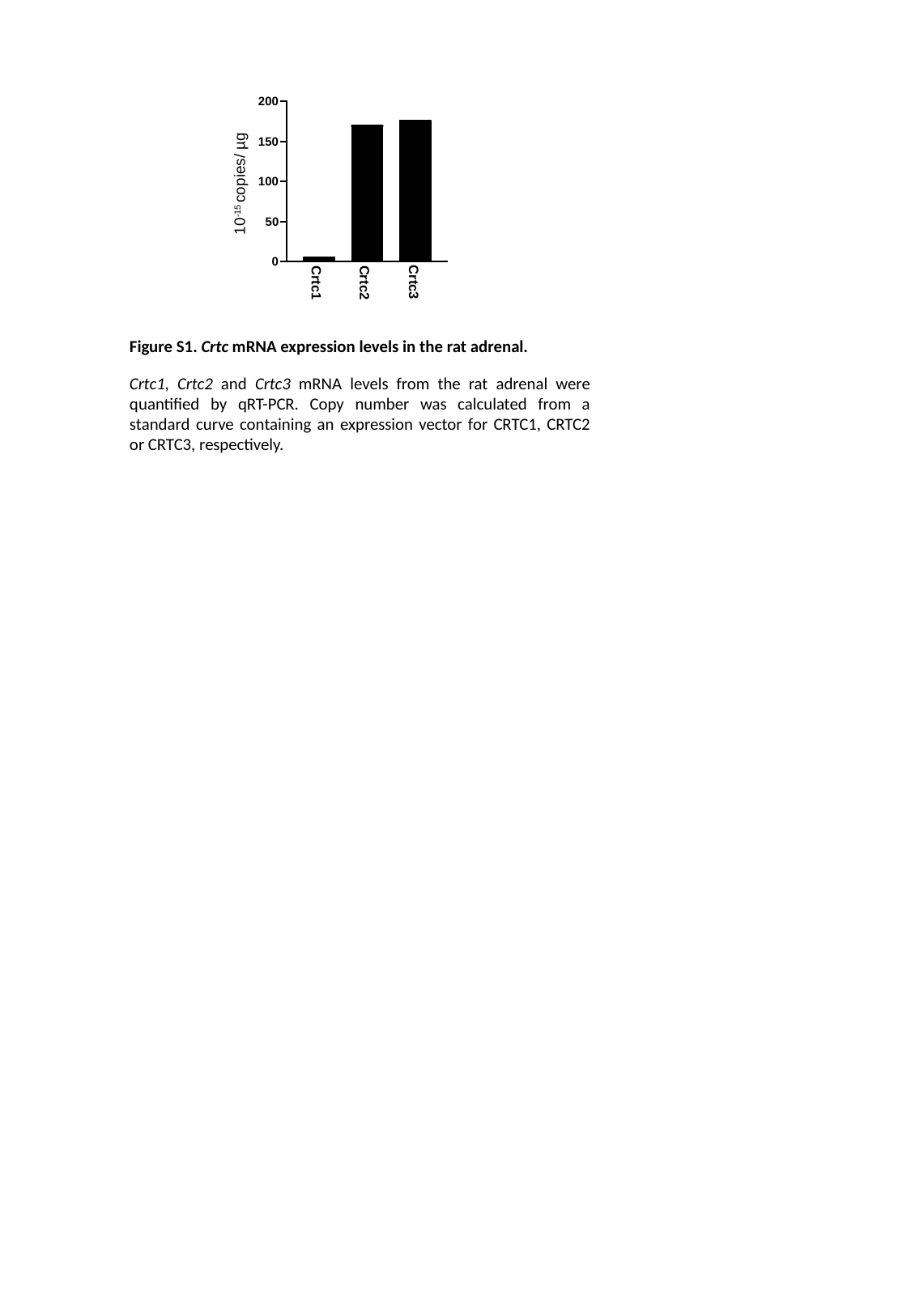

10-15 copies/ µg
Crtc3
Crtc2
Crtc1
Figure S1. Crtc mRNA expression levels in the rat adrenal.
Crtc1, Crtc2 and Crtc3 mRNA levels from the rat adrenal were quantified by qRT-PCR. Copy number was calculated from a standard curve containing an expression vector for CRTC1, CRTC2 or CRTC3, respectively.
